# Data on Swiss consumers’ perception of different types of sustainability levies, agriculture and willingness to choose suboptimal potatoes in different settings

**DOI:** 10.1016/j.dib.2025.111551

**Published:** 2025-04-11

**Authors:** Jeanine Ammann, Gabriele Mack, Rita Saleh

**Affiliations:** a Agroscope, Research Group Economic Modelling and Policy Analysis, Ettenhausen, Switzerland; b Agroscope, Research Group Socioeconomics, Ettenhausen, Switzerland

**Keywords:** Food policy, Trust, Animal welfare, Social sustainability, Suboptimal food, Food waste

## Abstract

We present representative survey data from 481 Swiss consumers. Data were collected in the German-speaking parts of Switzerland in February and March 2024. The survey includes three independent main parts.

In a first part, we collected qualitative and quantitative data on participants’ perception of Swiss agriculture and farmers. Specifically, participants’ trust in crop and livestock production farmers and their perceived knowledge about production methods and their affect towards farmers was assessed.

In a second part, we collected quantitative data on participants’ preference for different sustainability levies. For this, six different products were used (i.e., fresh/processed vegetables, dairy, and meat). For each of these six products, participants were shown four levy options from which they had to choose the one that they found most appealing. For vegetables, the options were: (A) reduction of risks related to plant protection products, (B) more support for local farmers, (C) support for environmental sustainability, and (D) sustainability projects in general. For the animal products, option (A) was an increase in animal welfare, whilst options (B), (C) and (D) were the same as for the vegetable products.

In a third part, we collected qualitative and quantitative data on participants preferences for suboptimal or optimal potatoes. Here, a 2 × 2 experimental design (setting × information) was used. This means that participants were presented with either a supermarket or farm shop setting and with or without food waste information. Participants then chose between two potatoes: optimal potato A, suboptimal potato B, or neither. Both potatoes were equally expensive.

Further, we collected personal information about participants such as gender, age, education level and how they placed themselves regarding their political orientation on a left-right scale. We further collected behavioural data including diet, that is, milk and meat consumption frequency as well as shopping behaviour, where we asked participants where they usually did their grocery shopping. At the end of the survey, we used existing and new scales to measure participants’ perception of farmers, health consciousness and environmental attitudes. Before collecting this data, ethical approval was obtained from the Agroscope ethical commission (application EK-AGS-2024-N-01).

Specifications Table

**Table:** 

Subject	Applied Psychology, Sociology, Food Science.
Specific subject area	*Perception of farmers and agriculture, preference for different types of policy measures and willingness to choose suboptimal potatoes in different experimental settings.*
Type of data	Table, Image, Figure CSV file (semicolon delimited), survey (PDF) and codebook (PDF) Raw and cleaned.
Data collection	Participants were recruited by Bilendi AG (ISO-certified panel provider) and the data were collected through an online survey (accessible from computer and phone) implemented with Tivian. Data collection took place in the German-speaking parts of Switzerland in February and March 2024. Quotas were used for age and gender. The survey took between 15 and 20 min to complete.
Data source location	Institution: Agroscope City/Town/Region: Ettenhausen, Tänikon Country: Switzerland*.*
Data accessibility	Repository name: Zenodo Data identification number: 10.5281/zenodo.13736435 Direct URL to data: https://zenodo.org/records/13736436 [1] Instructions for accessing these data: data are freely available
Related research article	*Ammann, J., Liechti, C., Mack, G., Saleh, R. (2025). A food waste information-framing can help promote purchase of suboptimal potatoes.*https://doi.org/10.1016/j.foodqual.2024.105338*[**2**].*

## 1 Value of the Data

• The data on public trust in farmers are important when designing agricultural policies
• The data on participants’ preferences for different sustainability levies can inform measures to promote behaviour change
• The data on participants’ preference for suboptimal potatoes is important for practitioners trying to promote suboptimal potatoes
• The survey contains different methodological approaches which can be used by researchers or teachers
• The data can help farmers and consumers gain more awareness regarding different sustainability issues in agriculture
• Understanding consumer preferences can help farmers tailor their pricing strategies to better meet customer expectations

## 2 Background

The first part of the study on public trust in farmers builds on previous research assuming that a lack of public trust in farmers can impede public acceptance of farming technologies [3] and hence farmers’ adoption of these technologies. It can also lead to stricter and unfounded regulations on farming practices, thereby limiting farmers and even impacting the viability and sustainability of farms [4]. Therefore, the study aimed to examine public image of farmers and the agriculture sector.

The second part of the survey builds on research identifying taxes as efficient measures to facilitate sustainable behaviour change but tending to be unpopular [5]. Sustainability levies, which are a special type of tax, are less investigated and could come with fewer prejudices. Which is why they are investigated more closely in this study.

The third part of the survey builds on research identifying that due to a focus on aesthetics, huge quantities of produce are discarded along the food value chain even before reaching the supermarket [6]. One example is fresh potatoes, where only around 50 % of the potatoes produced reach the consumer [7]. Therefore, this study tests the effectiveness of information framings to make suboptimal potatoes more appealing.

## 3 Data Description

Data were collected through an online survey. Participant recruitment was done by a professional panel provider (Bilendi AG). We used quotas for gender (50 % women) and age (33 % aged 18–35, 33 % aged 36–54, and 33 % aged 55–75). In total, we aimed for 500 participants. A total of 525 participants completed our survey. We excluded participants who took less than half the median of all participants to complete the survey, that is, <362 s, as we assumed that for these observations, data quality was not sufficient. This data cleaning procedure led to a final sample size of 481 participants. Refer to Table 1 for an overview on the final sample. The dataset in wide format after data cleaning (cleaned; CSV), the survey in two languages (PDF) and the codebook describing the variables (PDF) are freely available online from Zenodo [1]. All variables and the corresponding questions are explained in the codebook (see Supplementary Materials).

**Table 1:** Sample description (*N* = 481).

	%	Mean	SD
Gender (women)	51.1		
Age		46.9	15.7
Education			
No education, in education	0.2		
Compulsory school	4.2		
Vocational apprenticeship / vocational college / commercial (secondary) school	46.6		
(Vocational) baccalaureate	9.1		
Higher technical or vocational education	18.9		
University of applied sciences or university of education	10.8		
University	10.2		
Political orientation		52.0	19.8
Current place of residence			
Very rural	13.7		
Rather rural	34.5		
Suburban	28.3		
Rather urban	15.6		
Very urban	7.9		
Meat and meat product consumption frequency			
Several times a day	2.3		
Daily	15.0		
4 to 6 times per week	31.8		
1 to 3 times a week	36.8		
1 to 3 times a month	7.1		
Rarely	2.9		
Never	4.2		
Milk and dairy consumption frequency			
Several times a day	11.0		
Daily	46.6		
4 to 6 times per week	21.2		
1 to 3 times a week	15.8		
1 to 3 times a month	1.9		
Rarely	2.7		
Never	0.8		
Shopping at farm shops / markets			
Rarely or never shops at farm shops / markets	55.5		
Regularly shops at farm shops / markets	44.5		

## 4 Experimental Design, Materials and Methods

We collected survey data in Switzerland in February and March 2024. The survey was run with the survey software Tivian and the link was sent to participants. They were able to access it online or by phone. The survey consisted of twelve distinct parts as described in the following.

**Part 1: Introduction and consent**

**Part 2: Personal information**

Personal information (see Table 1)
  a. Age
  b. Gender
  c. Education level
    i. No education, in education
    ii. Compulsory school
    iii. Vocational apprenticeship / vocational college / commercial (secondary) school
    iv. (Vocational) baccalaureate
    v. Higher technical or vocational education
    vi. University of applied sciences or university of education
    vii. University
  d. Political orientation
  e. Current place of residence
    i. Very rural
    ii. Rather rural
    iii. Suburban
    iv. Rather urban
    v. Very urban
  f. Meat and meat product consumption frequency
    i. Several times a day
    ii. Daily
    iii. 4–6 times per week
    iv. 1–3 times per week
    v. 1–3 times per month
    vi. Rarely
    vii. Never
  g. Milk and dairy consumption frequency
    i. Several times a day
    ii. Daily
    iii. 4–6 times per week
    iv. 1–3 times per week
    v. 1–3 times per month
    vi. Rarely
    vii. Never
  h. Grocery shopping channels and frequency

**Part 3: Agriculture (mixed-method)**

 Spontaneous associations with “agriculture in Switzerland” (qualitative)
  Hedonic rating of the association (quantitative)
 Spontaneous associations with “farmers in Switzerland” (qualitative)
  Hedonic rating of the association (quantitative)
 Fruit and vegetable farmers
  Trust in vegetable and fruit growers to produce healthy food
  Trust in vegetable and fruit growers to take good care of the environment
  How transparent are vegetable and fruit growers with regard to cultivation methods
  How much knowledge do you have in terms of vegetable and fruit production
 Milk and meat production
  Trust in livestock farmers to produce healthy food
  Trust in livestock farmers to take good care of the environment
  Trust in livestock farmers to take good care of their animals
  How transparent are livestock farmers with regard to their production methods
  How much knowledge do you have in terms of livestock farming
 Commitment and support
  Practical experience in agriculture
  Family members working in agriculture
  Knowing someone personally who works in agriculture
  Knowing no one who works in agriculture
  Should financial support for agriculture change in the future

**Part 4: Sustainability levy**

 Fresh vegetables / processed vegetables
 Milk / dairy products
 Meat / processed meat products

**Part 5: Potato experiment (mixed-method)**

• Control / producer
• Food waste framing / producer
• Control / supermarket
• Food waste framing / supermarket

**Part 6: Personal attitudes**

1. Perception of farmers
2. Health consciousness
3. Environmental Attitudes Inventory (EAI)
  a. Scale 4
  b. Scale 8

**Part 7: Thank you and end of the survey**

The different parts and their content are described in more detail in the following.

**Part 1: Introduction and consent**

In the first part of the survey, participants were briefly informed about the contents of the survey and provided their informed consent. They were informed that ethic approval was obtained from the Agroscope ethical commission (application EK-AGS-2024-N01). Further, we informed them that they were free to quit the survey at any time without having to give a reason.

**Part 2: Personal information**

In the second part of the survey, personal information was obtained. This included participants’ age, gender, education level and current place of residence. When it came to their level of education, the participants indicated the highest qualification they had achieved, choosing from seven options (from ‘no education / in education’ to ‘university’). Compulsory school in Switzerland takes around 11 years.

Next, from five options, the participants selected the one that they felt best described their current place of residence (from very urban to very rural). The five response categories were chosen in accordance with previous research and with the terms used by the Swiss Statistical Office [8]. The breakdown of municipalities used by the Swiss Statistical Office is based on the so-called Urban/Rural Typology 2012, which separates two urban areas, that is, core cities and other urban municipalities, an intermediary settlement type with both urban and rural characteristics, and rural areas.

Next, participants were asked about their behaviour, including political orientation and shopping. For participants’ political orientation, we asked them to place themselves on an interactive slider from left (0) to right (100), as done similarly by other studies including Eurobarometer [9]. The middle of the scale was marked to help participants orient themselves. However, no start position was given for the curser in order not to influence participants. The curser only appeared after they clicked on the interactive slider. Meat and dairy consumption frequency were measured on a 6-point response scale from 1 (rarely or never) to 6 (multiple times per day).

**Part 3: Agriculture (mixed-method)**

In this part, participants were instructed indicate their associations with agriculture by writing down the first words that come to their mind when they think of “agriculture” in general. A text field was provided for this purpose. To understand whether their associations were positive or negative, they were subsequently asked to rate their feelings regarding the association they wrote down earlier using a slider scale. This interactive slider displayed verbal anchors at either end (0 = very negative to 100 = very positive) and the centre was visually indicated by a line. Next, participants indicated their associations and feelings regarding “Swiss farmers”.

To capture participants’ perceptions of farmers engaged in crop and animal production, they were asked about their trust levels using three items. In the first item, they were asked how much trust they have in crop farmers (i.e., fruit and vegetable growers) and then in livestock farmers (i.e., milk and meat producers) to produce good food for the Swiss population. In a second item, they were asked about their trust in these farmers to care for the environment. The third item asked them about their trust in these farmers to being transparent with their farming practices. For the animal production farmers, an additional item was added related to trust to care for animal welfare. For all of these items, participants indicated their trust levels on a slider scale ranging from 0 = no trust at all to 100 = completely trustworthy.

Further, participants rated their own knowledge levels of crop and animal production on a slider scale ranging from 0 = *I* know very little about it to 100 = *I* know a lot about it. Finally, participants indicated their thoughts on how financial support of agriculture should change in the future using a slider scale from 0 = support should be less, 50 = it should remain the same to 100 = support should increase. At the end of this part, they selected if they have practical experience working in the agriculture sector themselves or not, or have a family member or know someone who works in agriculture or not.

**Part 4: Sustainability levy**

In a next experimental part, participants assessed different framings of a hypothetical sustainability levy. They were informed that the supermarket where they most frequently did their grocery shopping used part of the selling price of a specific product for sustainability projects. The participants were then presented with six different product categories one at the time, and each product was combined with four options of a sustainability levy. For each product, the participants were asked to choose one of the four levy options.

The product categories chosen for the experimental part included fresh and processed vegetables, dairy, and meat. For the levy options, different areas of sustainability were covered. Specifically, levy option A captured the reduction of risks of plant protection products for vegetables. For the animal-based product, animal welfare was used instead. Levy option B included the support of local farmers as a measure of social sustainability for all food categories. For levy option C, a reduction in the ecological footprint was included as a measure of environmental sustainability. Finally, levy option D included sustainability as an umbrella term without a specific description.

**Part 5: Potato experiment (mixed-method)**

In the potato experiment, the perception of optimal and suboptimal potatoes was examined. For the suboptimal potatoes, potatoes with visible scab and slightly larger size were selected. This corresponds to the current situation in Switzerland with regard to potatoes that tend to be difficult to sell.

The first, quantitative part of the potato experiment followed a 2 × 2 design (setting × information). Participants were presented with either a supermarket or farm shop *setting.* Further, the description was shown with or without additional food waste *information*. Depending on the setting, participants read the following instructions: “Imagine you want to buy firm cooking potatoes. You find the following two product variants in a [farm shop]/[supermarket]. Which product would you choose?” Participants were shown a description including a picture of two potatoes, A and B. Potato A corresponds to what is commonly found in supermarkets in Switzerland. Potato B has a suboptimal appearance with visible marks of common scab.

**Fig. 1:**
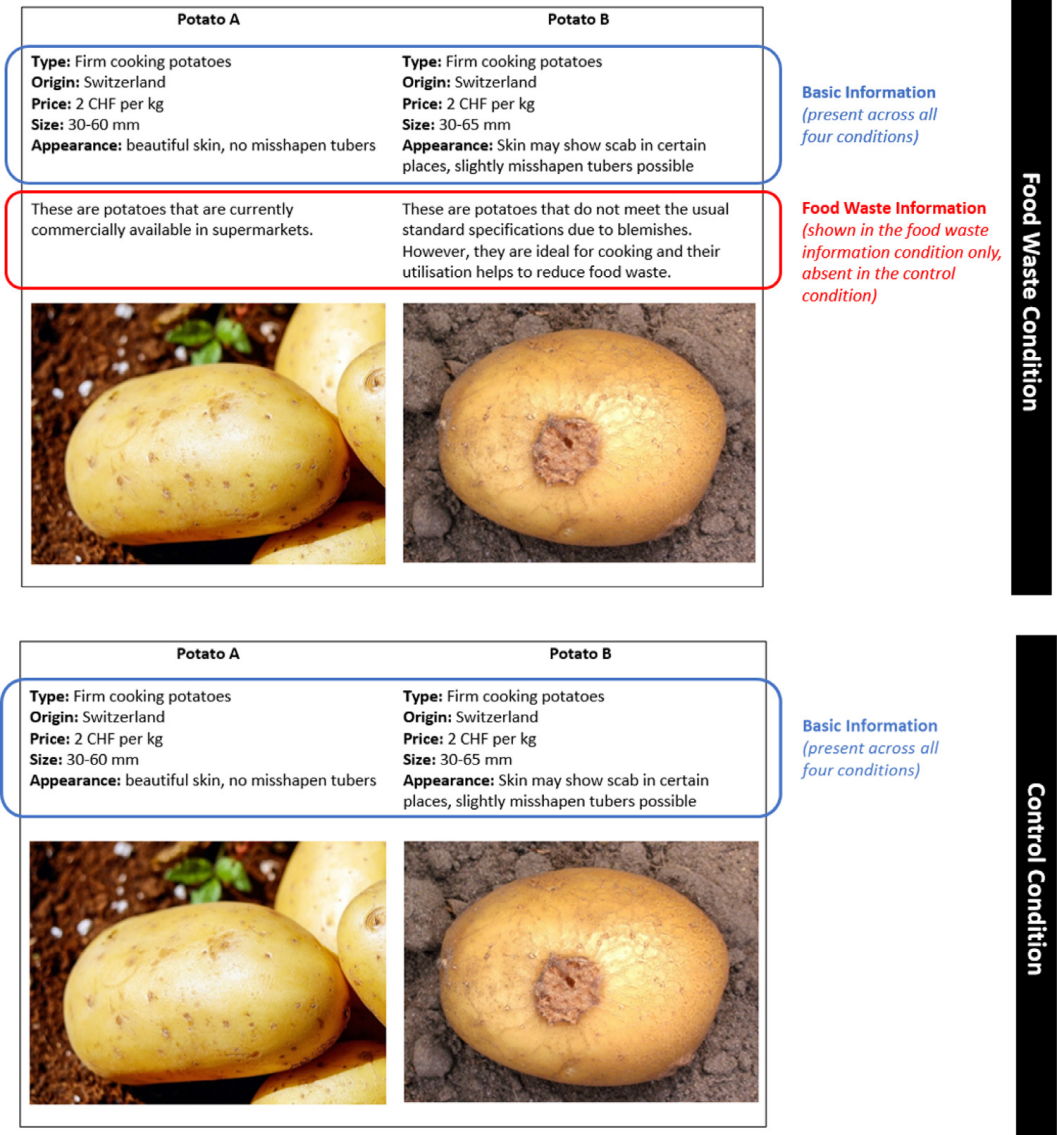
**Experimental design showing the information provided to participants across experimental conditions.**^1^

For the food waste information condition, additional information was added for each of the potatoes. For potato A, it was explained that these were potatoes currently commercially available in supermarkets. For potato B, it was explained that these potatoes did not meet the usual standard specifications due to blemishes. In the control condition, no additional information describing the potatoes was added, and there was no mention of food waste (see Fig. 1).

As a result, there were four experimental conditions: (1) farm shop/control, (2) farm shop/food waste information, (3) supermarket/control and (4) supermarket/food waste information. All participants, irrespective of the experimental condition to which they had been assigned, chose between the three options of potato A (optimal appearance), potato B (suboptimal appearance), or neither. Both potatoes were offered at the same price to control for price effects.

After choosing a potato, in a second, qualitative part, participants were asked to explain their choice in a few words. For this, they were offered a text field.

**Part 6: Personal attitudes**

In terms of personal attitudes, participants were asked to rate ten items on perception of farmers. Five items have been tested in previous studies [10] and another five items were tested for the first time. The latter items were dropped from the perceptions of farmers scale as they did not perform well with the overall scale. Hence, the scale was built using the means of the five items previously used in other studies [10]. The reliability of the scale was good (α = 0.84, *M* = 5.41, SD = 1.00). Sample items include “I am generally positive about farmers”.

Participants’ health consciousness was measured with four items according to Dohle et al. (2014) [11]. Each item was rated for agreement on a scale from 1 (do not agree at all) to 6 (totally agree). The reliability of the scale was good (α = 0.80, *M* = 4.58, SD = 0.91). Sample items include “My health is dependent on how and what I eat”.

Participants’ environmental attitudes were assessed using 10 items from scale 4 (conservation motivated by anthropocentric concern) and 10 items of scale 8 (personal conservation behaviour) of the Environmental Attitudes Inventory by Milfont and Duckitt [12]. Each item was rated for agreement on a scale from 1 (do not agree at all) to 7 (totally agree). The reliability of scale 4 was good (α = 0.62, *M* = 3.2, SD = 1.3). The reliability of scale 8 was good (α = 0.83, *M* = 5.4, SD = 1.0). Sample items include “One of the best things about recycling is that it saves money” or “Whenever possible, I take a short shower in order to conserve water”.

**Part 7: Thank you and end of the survey**

In the final part of the survey, participants were given the possibility to write down any comments if they wished to do so. After that, we thanked participants for their participation and they were instructed to close the survey.

## Limitations

When working with this data, researchers need to keep in mind that it was obtained from the German-speaking parts of Switzerland. Future research is required to see whether these findings can be transferred to the other language regions. For the purchase situations, it has to be considered that participants were faced with simplified scenarios. More research is needed to test whether the results are similar when participants face more realistic purchase settings.

## Ethics Statement

All participants involved in the study provided their written, informed consent to participate. Participation was voluntary and could be withdrawn at any time. Participants remained anonymous and their responses were dealt with in confidence. Ethical approval was obtained from the Agroscope ethical commission (application EK-AGS-2024-N-01).

## CRediT authorship contribution statement

**Jeanine Ammann:** Conceptualization, Investigation, Data curation, Writing – original draft, Project administration. **Gabriele Mack:** Conceptualization, Writing – review & editing. **Rita Saleh:** Conceptualization, Investigation, Data curation, Writing – review & editing.

## Data Availability

ZenodoDataset on consumers' perception of different types of sustainability levies, Swiss agriculture and farmers and willingness to choose suboptimal potatoes in different settings (Original data).

## Appendix Supplementary Materials

Image, application 1

## Footnotes

1 Picture source for potato A: pixabay.com (https://pixabay.com/de/photos/kartoffeln-gem%C3%BCse-lebensmittel-1585060/) Picture source for potato B: wikipedia.org (https://upload.wikimedia.org/wikipedia/commons/1/17/Aardappelschurft_%28Streptomyces_scabies_on_potato%29.jpg).
