# Supplementary material for: Data on Swiss consumers’ perception of different types of sustainability levies, agriculture and willingness to choose suboptimal potatoes in different settings
Source: Data Brief. 2025 Apr 11;60:111551. doi: 10.1016/j.dib.2025.111551 (PMC12175247; doi:10.1016/j.dib.2025.111551)
Supplement: Supplementary file 1 [file mmc1.docx]

Codebook

Project: Sustainability Levy

| **Variable name data** | **Variable name DIB** | **German (original)** | **English (translation)** |
| --- | --- | --- | --- |
|  | **Introduction and Consent** | **Survey Part 1: Einverständnis** | **Survey Part 1: Consent** |
|  |  | Warum werde ich gebeten, an dieser Forschungsstudie teilzunehmen?  Wir laden Sie ein, an einer Studie teilzunehmen, in der wir mehr über Ihre Einstellung zu Landwirtschaft und Ernährung erfahren möchten. Sie müssen mindestens 18 Jahre alt sein, um an der Umfrage teilnehmen zu können.  Was ist der Zweck dieser Studie?  Die Umfrage soll neue wissenschaftliche Erkenntnisse über die Wahrnehmung der Landwirtschaft und Ernährung in der Schweiz hervorbringen. Diese Studie wird von Agroscope, dem Kompetenzzentrum des Bundes für die landwirtschaftliche Forschung, durchgeführt  Wie viel Zeit muss ich investieren?  Die Teilnahme an der Umfrage dauert ca. 15-20 Minuten.  Was ist der genaue Inhalt der Umfrage?  Sie werden gebeten, verschiedene Lebensmittel zu bewerten und Aspekte der Landwirtschaft zu gewichten. Sie beantworten zudem einige soziodemografische Fragen zu Ihrer Person.  Was passiert, wenn ich nicht teilnehmen möchte?  Ihre Teilnahme an dieser Studie ist freiwillig. Sie können sich entscheiden, nicht an dieser Studie teilzunehmen. Zudem können Sie die Befragung jederzeit abbrechen.  Könnte mir die Teilnahme an dieser Studie irgendwie schaden?  Diese Umfrage enthält keine Fragen, die Ihnen ein Risiko bringen oder Ihnen Unbehagen bereiten könnten. Sie können jedoch die Umfrage an jeder Stelle abbrechen.  Was geschieht mit den für die Forschung gesammelten Informationen?  Es werden keine direkten persönlichen Merkmale erfasst. Ihre Angaben werden im Rahmen der gesetzlichen Bestimmungen stets vertraulich behandelt. Die Ergebnisse und die Daten der Forschungsstudie können veröffentlicht werden, Ihre Identität bleibt jedoch immer anonym. Ihre Daten werden Forschenden von Agroscope zugänglich sein.  Mit wem kann ich sprechen?  Fragen zu dieser Studie können gerne an die Studienleitung (Dr. Jeanine Ammann, jeanine.ammann@agroscope.admin.ch oder Dr. Rita Saleh, rita.saleh@agroscope.admin.ch) gerichtet werden.  Zustimmung  Wenn Sie eine Kopie dieser Zustimmung für Ihre Unterlagen benötigen, können Sie sie ausdrucken.  Wenn Sie teilnehmen möchten, klicken Sie nun bitte auf die Schaltfläche "Ich stimme zu" und Sie werden zur Umfrage weitergeleitet.  Wenn Sie nicht an dieser Studie teilnehmen möchten, wählen Sie bitte "Ich stimme nicht zu" oder wählen Sie X in der Ecke Ihres Browsers.  Mit Ihrer Zustimmung, bestätigen Sie, dass Sie die obige Erklärung gelesen haben und die Möglichkeit hatten, Fragen zu stellen und Bedenken zu äussern. Sie bestätigen, dass Sie den Zweck der Studie sowie die damit einhergehenden potenziellen Risiken verstehen. Sie bestätigen, dass Ihre Teilnahme freiwillig ist und dass Sie mit Ihrer Zustimmung auf keine Rechte verzichten. Sie bestätigen, dass Sie mindestens 18 Jahre alt sind. Sie können die Studie jederzeit abbrechen. | Why am I asked to take part in this study?  We invite you to take part in a study in which we would like to find out more about your attitude to agriculture and food. You must be at least 18 years old to take part in the survey.  What is the aim of this study?  The survey is intended to provide new scientific insights into the perception of agriculture and nutrition in Switzerland. This study is being conducted by Agroscope, the federal government's centre of excellence for agricultural research.  How much time do I need?  Participation in our study will require around 15-20 minutes.  What is the content of the survey?  You will be asked to evaluate various foods and prioritise aspects of agriculture. You will also answer some socio-demographic questions about yourself.  What happens if I decide that I do not wish to participate?  Your participation in this study is voluntary. You can decide not to take part in this study. You can also cancel the survey at any time.  Is there any risk related to participation in this study?  This survey does not contain any questions that could put you at risk or make you feel uncomfortable. However, you can cancel the survey at any point.  What happens with the data collected through this research?  No direct personal characteristics are recorded. Your details will always be treated confidentially in accordance with legal regulations. The results and data of the research study may be published, but your identity will always remain anonymous. Your data will be accessible to Agroscope researchers.  Who can I contact?  Questions about this study can be directed to the study director (Dr Jeanine Ammann, jeanine.ammann@agroscope.admin.ch or Dr Rita Saleh, rita.saleh@agroscope.admin.ch).  Consent  If you need a copy of this consent for your records, you can print it out.  If you wish to participate, please click on the ‘I agree’ button and you will be redirected to the survey.  If you do not wish to participate in this study, please select ‘I do not consent’ or select X in the corner of your browser.  By giving your consent, you acknowledge that you have read the above statement and have had the opportunity to ask questions and express concerns. You confirm that you understand the purpose of the study and the potential risks involved. You confirm that your participation is voluntary and that you do not waive any rights by giving your consent. You confirm that you are at least 18 years old. You may withdraw from the study at any time. |
| V1 |  | Wenn Sie mit der obigen Erklärung einverstanden sind, klicken Sie auf «Ich stimme zu und beginne mit der Studie» und auf «weiter», um fortzufahren.  X Ich stimme zu und beginne mit der Studie [1]  X Ich stimme nicht zu und möchte nicht an der Studie teilnehmen. [0 -> ausgefiltert] | If you agree with the above statement, click on ‘I agree and start the study’ and on ‘continue’ to proceed.  X I agree and start the study [1]  X I do not agree and do not wish to participate [0 -> filter out] |
|  | **Personal Information** | **Teil 2** | **Part 2** |
|  |  | Nun folgen ein paar Fragen zu Ihrer Person. | Now a few questions about yourself. |
| v_79 | Age | Bitte geben Sie uns Ihr Geburtsjahr im Format JJJJ an (z.B. 1993). | Please enter your year of birth in the format YYYY (e.g. 1993). |
| v_78 | Gender | Bitte geben Sie uns Ihr Geschlecht an.   - Mann [1] - Frau [2] - Anderes [3] - Möchte nicht antworten [4] | Please indicate your gender.   - Man [1] - Woman [2] - Other [3] - Do not want to answer [4] |
| v_105 | Education | Mein höchster, abgeschlossener Berufs- oder Schulabschluss ist:   - Kein Abschluss / in Ausbildung [1] - Obligatorische Schule [2] - Berufslehre / Berufsfachschule / Handels(mittel)schule [3] - Maturität / Berufsmaturität [4] - Höhere Fach- oder Berufsausbildung [5] - Fachhochschule oder pädagogische Hochschule [6] - Universität / ETH [7] | My highest completed vocational or school qualification is:   - No degree / in training [1] - Compulsory school [2] - Vocational apprenticeship / Vocational school / Trade (secondary) school [3] - Matura / vocational baccalaureate [4] - Higher technical or vocational training [5] - University of applied sciences or teacher training college [6] - University / ETH [7] |
| v_106 | Political orientation | Wo ordnen Sie sich auf einer politischen links-rechts Skala ein?  Bitte klicken Sie in den Balken, um Ihre Antwort abzugeben.  [interactive slider scale from 0 (sehr links) – 50 (Mitte) – 100 (sehr rechts)] | Where do you place yourself on a political left-right scale?  Please click in the bar to submit your answer.  [interactive slider scale from 0 (far left) - 50 (center) - 100 (far right)] |
| v_107 | Current place of residence | Was trifft am ehesten auf Ihren aktuelle Wohngegend zu? (analog Ammann et al., 2023)   - Sehr ländlich (1) - Eher ländlich (2) - Vorstädtisch (3) - Eher städtisch (4) - Sehr städtisch (5) | What is most likely to apply to your current residential area? (similar to Ammann et al., 2023)   - Very rural (1) - Rather rural (2) - Suburban (3) - Rather urban (4) - Very urban (5) |
| v_7 | Meat and meat product consumption frequency | Wie oft konsumieren Sie Fleisch und Fleischprodukte? (in Anlehnung an Ammann et al., 2023; Michel et al., 2021)  *(Hinweis: Hier ist nur Fleisch gemeint und keine pflanzlichen Fleischersatzprodukte)*   - Mehrmals täglich [1] - Täglich [2] - 4-6 mal pro Woche [3] - 1-3 mal pro Woche [4] - 1-3 mal pro Monat [5] - Selten [6] - Nie [7] | How often do you consume meat and meat products? (inspired by Ammann et al., 2023; Michel et al., 2021)  *(Note: Only meat is meant here and no plant-based meat substitutes)*   - Several times a day [1] - Daily [2] - 4-6 times per week [3] - 1-3 times per week [4] - 1-3 times per month [5] - Rare [6] - Never [7] |
| v_8 | Milk and dairy consumption frequency | Wie oft konsumieren Sie Milch und Milchprodukte?  *(Hinweis: Hier ist nur Milch gemeint und keine pflanzlichen Milchersatzprodukte)*   - Mehrmals täglich [1] - Täglich [2] - 4-6 mal pro Woche [3] - 1-3 mal pro Woche [4] - 1-3 mal pro Monat [5] - Selten [6] - Nie [7] | How often do you consume milk and dairy products?  *(Note: Only milk is meant here and no plant-based milk substitutes)*   - Several times a day - Daily - 4-6 times per week - 1-3 times per week - 1-3 times per month - Rare - Never |
|  | Grocery shopping channels and frequency | Wie oft kaufen Sie Lebensmittel über folgende Verkaufskanäle:   - Mehrmals pro Woche [6] - 1 mal pro Woche [5] - Alle zwei Wochen [4] - 1 mal pro Monat [3] - selten [2] - gar nie [1] | How often do you buy food through the following sales channels:   - Several times a week [6] - 1 time per week [5] - Every two weeks [4] - 1 time per month [3] - rare [2] - never [1] |
| v_9 |  | Online (z.B. Farmy, Coop at Home, Migros Online, etc.) | Online (e.g. Farmy, Coop at Home, Migros Online, etc.) |
| v_10 |  | Im Supermarkt (z.B. Migros, Coop etc.) | In the supermarket (e.g. Migros, Coop etc.) |
| v_11 | Shopping at farm shops / markets | Im Direktverkauf auf dem Hof / auf dem Markt | Direct sales on the farm / at the market |
| v_14 |  | - Andere: ___________ | - Other: ___________ |
| v_15 |  | - Andere: ___________ [Textfeld] | - Other: ___________ [textfield] |

| **Variable name data** | **Variable name DIB** | **German (original)** | **English (translation)** |
| --- | --- | --- | --- |
|  | **Agriculture (mixed-method)** | **Teil 3** | **Part 3** |
|  |  | Nun folgen ein paar Fragen zum Thema Landwirtschaft. | Next, we would like to ask you a few questions about agriculture. |
| v_16 | Spontaneous associations with «agriculture in Switzerland” | Was kommt Ihnen spontan als erstes in den Sinn, wenn Sie an die Landwirtschaft in der Schweiz denken? Bitte notieren Sie den ersten Gedanken oder das erste Bild, das Ihnen dazu einfällt (bitte nur einen Begriff notieren).  [Freitext] | What is the first thing that spontaneously comes to mind when you think of agriculture in Switzerland? Please write down the first thought or image that comes to mind (please note only one term).  [Free text] |
| v_109 | Hedonic rating | Wenn Sie an [hier steht die zuvor gegebene Antwort] denken, welche Gefühle verbinden Sie damit?  [interactive slider scale: 0 (sehr negativ) – 50 (neutral) – 100 (sehr positiv)] | When you think of [here is the answer you gave before], what feelings do you associate with it?  [interactive slider scale: 0 (very negative) - 50 (neutral) - 100 (very positive)] |
| v_110 | Spontaneous associations with «farmers in Switzerland” | Was kommt Ihnen spontan als erstes in den Sinn, wenn Sie an Landwirtinnen und Landwirte in der Schweiz denken? Bitte notieren Sie den ersten Gedanken oder das erste Bild, das Ihnen dazu einfällt (bitte nur einen Begriff notieren).  [Freitext] | What is the first thing that spontaneously comes to mind when you think of farmers in Switzerland? Please write down the first thought or image that comes to mind (please note only one term).  [Free text] |
| v_111 | Hedonic rating | Wenn Sie an [hier steht die zuvor gegebene Antwort] denken, welche Gefühle verbinden Sie damit?  [interactive slider scale: 0 (sehr negativ) – 50 (neutral) – 100 (sehr positiv)] | When you think of [here is the answer you gave before], what feelings do you associate with it?  [interactive slider scale: 0 (very negative) - 50 (neutral) - 100 (very positive)] |

|  |  | **Obst- und Gemüsebauern** | **Fruit and vegetable growers** |
| --- | --- | --- | --- |
| v_31 | Trust in vegetable and fruit growers to produce healthy food | Wie viel Vertrauen haben Sie in Gemüse- und Obstbauern in der Schweiz, dass sie gesunde Lebensmittel für die Bevölkerung produzieren?  interactive slider scale: 0= gar kein Vertrauen, 100 = sehr viel Vertrauen | How much trust do you have in vegetable and fruit growers in Switzerland that they produce healthy food for the population?  interactive slider scale: 0= no trust at all, 100 = very much trust |
| v_32 | Trust in vegetable and fruit growers to take good care of the environment | Wie viel Vertrauen haben Sie in Gemüse- und Obstbauern in der Schweiz, dass sie sich gut um die Umwelt kümmern?  interactive slider scale: 0= gar kein Vertrauen, 100 = sehr viel Vertrauen | How much trust do you have in vegetable and fruit growers in Switzerland that they are taking good care of the environment?  interactive slider scale: 0= no trust at all, 100 = very much trust |
| v_33 | How transparent are vegetable and fruit growers with regard to cultivation methods | Wie transparent sind Ihrer Meinung nach Gemüse- und Obstbauern in der Schweiz bezüglich Ihrer Anbaumassnahmen (wie Gemüse und Früchte auf dem Hof angebaut werden)?  interactive slider scale: 0= gar nicht transparent / 100 = sehr transparent | In your opinion, how transparent are vegetable and fruit growers in Switzerland with regard to their cultivation measures (how vegetables and fruit are grown on the farm)?  interactive slider scale: 0= not transparent at all / 100 = very transparent |
| v_34 | How much knowledge do you have in terms of vegetable and fruit production | Wie schätzen Sie Ihr eigenes Wissen zur Gemüse- und Obstproduktion ein?  interactive slider scale: 0 = darüber weiss ich sehr wenig, 100 = darüber weiss ich sehr viel | How would you rate your own knowledge of vegetable and fruit production?  interactive slider scale: 0 = I know very little about this, 100 = I know a lot about this |
|  |  | **Milch- und Fleischproduktion** | **Milk and meat production** |
| v_35 | Trust in livestock farmers to produce healthy food | Wie viel Vertrauen haben Sie in Tierhalter (Milch- und Fleischproduzenten) in der Schweiz, dass sie gesunde Lebensmittel für die Bevölkerung produzieren?  interactive slider scale: 0= gar kein Vertrauen, 100 = sehr viel Vertrauen | How much trust do you have in livestock farmers (milk and meat producers) in Switzerland that they produce healthy food for the population?  interactive slider scale: 0= no trust at all, 100 = very much trust |
| v_36 | Trust in livestock farmers to take good care of the environment | Wie viel Vertrauen haben Sie in Tierhalter (Milch- und Fleischproduzenten) in der Schweiz, dass sie sich gut um die Umwelt kümmern?  interactive slider scale: 0= gar kein Vertrauen, 100 = sehr viel Vertrauen | How much trust do you have in livestock farmers (milk and meat producers) in Switzerland that they take good care of the environment?  interactive slider scale: 0= no trust at all, 100 = very much trust |
| v_112 | How transparent are livestock farmers with regard to their production methods | Wie viel Vertrauen haben Sie in Tierhalter (Milch- und Fleischproduzenten) in der Schweiz, dass sie sich gut um die Tiere kümmern?  interactive slider scale: 0= gar kein Vertrauen, 100 = sehr viel Vertrauen | How much trust do you have in livestock farmers (milk and meat producers) in Switzerland that they take good care of the animals?  interactive slider scale: 0= no trust at all, 100 = very much trust |
| v_37 | How much knowledge do you have in terms of livestock farming | Wie transparent sind Ihrer Meinung nach Tierhalter (Milch- und Fleischproduzenten) in der Schweiz bezüglich Ihrer Praktiken (wie Milch und Fleisch auf dem Hof hergestellt werden)?  interactive slider scale: 0= gar nicht transparent / 100 = sehr transparent  Wie schätzen Sie Ihr eigenes Wissen zu tierischer Produktion ein?  interactive slider scale: 0 = darüber weiss ich sehr wenig, 100 = darüber weiss ich sehr viel | In your opinion, how transparent are livestock farmers (milk and meat producers) in Switzerland with regard to their practices (how milk and meat are produced on the farm)?  interactive slider scale: 0= not transparent at all / 100 = very transparent  How would you rate your own knowledge of animal production?  interactive slider scale: 0 = I know very little about this, 100 = I know a lot about this |
|  |  | **Engagement und Unterstützung** | **Commitment and support** |
|  |  | Bitte wählen Sie von den nachfolgenden Aussagen alle aus, die auf Ihre Situation zutreffen. | Please select all of the following statements that apply to your situation. |
| v_113 | Practical experience in agriculture | Ich habe selbst praktische Erfahrung in der Landwirtschaft (Ja [1]/nein [0]) | I have practical experience in agriculture (yes [1]/no [0]) |
| v_114 | Family members working in agriculture | Ich habe ein Familienmitglied, das in der Landwirtschaft tätig ist (Ja [1]/nein [0]) | I have a family member who works in agriculture (yes [1]/no [0]) |
| v_115 | Knowing someone personally who works in agriculture | Ich kenne jemanden persönlich (z.B. einen Freund, eine Nachbarin), der in der Landwirtschaft tätig ist (Ja [1]/nein [0]) | I know someone personally (e.g. a friend, a neighbour) who works in agriculture (yes [1]/no [0]) |
| v_116 | Knowing no one who works in agriculture | Ich kenne niemanden, der in der Landwirtschaft tätig ist ODER ich habe selbst keine Erfahrung in der Landwirtschaft (Ja [1]/nein [0], exklusive Antwort) | I do not know anyone who works in agriculture OR I have no experience in agriculture myself (yes [1]/no [0], exclusive answer) |
| v_40 | Should financial support for agriculture change in the future | Wie sollte sich Ihrer Meinung nach die finanzielle Unterstützung für die Landwirtschaft in Zukunft verändern?  [interactive slider scale from 0 (deutlich weniger Unterstützung) – 50 (gleich bleiben) – (deutlich mehr Unterstützung)] | How do you think financial support for agriculture should change in the future?  [interactive slider scale from 0 (significantly less support) - 50 (stay the same) - (significantly more support)] |
|  | **Sustainability Levy** | **Teil 4** | **Part 4** |
|  |  | Nun folgen ein paar Fragen zum Lebensmittelkauf. | Next, we would like to ask you a few questions about your food shopping behaviour. |
|  |  | Stellen Sie sich vor, dass im Supermarkt, wo Sie am häufigsten einkaufen, ein Teil des Verkaufspreises eines bestimmten Produkts für Nachhaltigkeitsprojekte verwendet wird. Nachfolgend sehen Sie jeweils eine Produktkategorie und vier mögliche Produktversionen, die alle gleich viel kosten. Bitte wählen Sie davon das Produkt, welches Sie am ehesten kaufen würden. | Imagine that in the supermarket where you shop most often, part of the sales price of a certain product is used for sustainability projects. Below you will see one product category and four possible product versions, all of which cost the same. Please select the product that you would be most likely to buy. |
|  |  | **Frisches Gemüse** | **Fresh vegetables** |
| v_41 |  | [1]: Ein Teil vom Verkaufspreis wird verwendet, um Projekte zur Reduzierung des Risikos von Pflanzenschutzmitteln zu fördern.  [2]: Ein Teil vom Verkaufspreis wird verwendet, um Projekte zur Unterstützung von Landwirtinnen und Landwirten in der Region zu fördern.  [3]: Ein Teil vom Verkaufspreis wird verwendet, um Projekte zur Reduzierung des ökologischen Fussabdrucks (z.B. CO2 Emissionen) der Lebensmittelproduktion zu fördern.  [4]: Ein Teil vom Verkaufspreis wird verwendet, um Projekte im Bereich der Nachhaltigkeit zu fördern. | [1]: A portion of the sales price is used to support projects to reduce the risk of pesticides.  [2]: A portion of the sales price is used to promote projects to support farmers in the region.  [3]: A portion of the sales price is used to support projects to reduce the ecological footprint (e.g. CO2 emissions) of food production.  [4]: A portion of the sales price is used to support sustainability projects. |
|  |  | **Verarbeitetes Gemüse (z.B. Tiefkühlware)** | **Processed vegetables (e.g. frozen goods)** |
| v_42 |  | [1]: Ein Teil vom Verkaufspreis wird verwendet, um Projekte zur Reduzierung des Risikos von Pflanzenschutzmitteln zu fördern.  [2]: Ein Teil vom Verkaufspreis wird verwendet, um Projekte zur Unterstützung von Landwirtinnen und Landwirten in der Region zu fördern.  [3]: Ein Teil vom Verkaufspreis wird verwendet, um Projekte zur Reduzierung des ökologischen Fussabdrucks (z.B. CO2 Emissionen) der Lebensmittelproduktion zu fördern.  [4]: Ein Teil vom Verkaufspreis wird verwendet, um Projekte im Bereich der Nachhaltigkeit zu fördern. | [1]: A portion of the sales price is used to support projects to reduce the risk of pesticides.  [2]: A portion of the sales price is used to promote projects to support farmers in the region.  [3]: A portion of the sales price is used to support projects to reduce the ecological footprint (e.g. CO2 emissions) of food production.  [4]: A portion of the sales price is used to support sustainability projects. |

|  |  | **Milch** | **Milk** |
| --- | --- | --- | --- |
| v_43 |  | [1]: Ein Teil vom Verkaufspreis wird verwendet, um Projekte zur Verbesserung des Tierwohls zu fördern.  [2]: Ein Teil vom Verkaufspreis wird verwendet, um Projekte zur Unterstützung von Landwirtinnen und Landwirten in der Region zu fördern.  [3]: Ein Teil vom Verkaufspreis wird verwendet, um Projekte zur Reduzierung des ökologischen Fussabdrucks (z.B. CO2 Emissionen) der Lebensmittelproduktion zu fördern.  [4]: Ein Teil vom Verkaufspreis wird verwendet, um Projekte im Bereich der Nachhaltigkeit zu fördern. | [1]: A portion of the sales price is used to support projects to improve animal welfare.  [2]: A portion of the sales price is used to promote projects to support farmers in the region.  [3]: A portion of the sales price is used to support projects to reduce the ecological footprint (e.g. CO2 emissions) of food production.  [4]: A portion of the sales price is used to support sustainability projects. |
|  |  | **Milchprodukte (z.B. Käse oder Joghurt)** | **Dairy products (e.g. cheese or yogurt)** |
| v_44 |  | [1]: Ein Teil vom Verkaufspreis wird verwendet, um Projekte zur Verbesserung des Tierwohls zu fördern.  [2]: Ein Teil vom Verkaufspreis wird verwendet, um Projekte zur Unterstützung von Landwirtinnen und Landwirten in der Region zu fördern.  [3]: Ein Teil vom Verkaufspreis wird verwendet, um Projekte zur Reduzierung des ökologischen Fussabdrucks (z.B. CO2 Emissionen) der Lebensmittelproduktion zu fördern.  [4]: Ein Teil vom Verkaufspreis wird verwendet, um Projekte im Bereich der Nachhaltigkeit zu fördern. | [1]: A portion of the sales price is used to support projects to improve animal welfare.  [2]: A portion of the sales price is used to promote projects to support farmers in the region.  [3]: A portion of the sales price is used to support projects to reduce the ecological footprint (e.g. CO2 emissions) of food production.  [4]: A portion of the sales price is used to support sustainability projects. |

|  |  | **Fleisch (z.B. Steak)** | **Meat (e.g. steak)** |
| --- | --- | --- | --- |
| v_45 |  | [1]: Ein Teil vom Verkaufspreis wird verwendet, um Projekte zur Verbesserung des Tierwohls zu fördern.  [2]: Ein Teil vom Verkaufspreis wird verwendet, um Projekte zur Unterstützung von Landwirtinnen und Landwirten in der Region zu fördern.  [3]: Ein Teil vom Verkaufspreis wird verwendet, um Projekte zur Reduzierung des ökologischen Fussabdrucks (z.B. CO2 Emissionen) der Lebensmittelproduktion zu fördern.  [4]: Ein Teil vom Verkaufspreis wird verwendet, um Projekte im Bereich der Nachhaltigkeit zu fördern. | [1]: A portion of the sales price is used to support projects to improve animal welfare.  [2]: A portion of the sales price is used to promote projects to support farmers in the region.  [3]: A portion of the sales price is used to support projects to reduce the ecological footprint (e.g. CO2 emissions) of food production.  [4]: A portion of the sales price is used to support sustainability projects. |
|  |  | **Verarbeitete Fleischprodukte (z.B. Würste)** | **Processed meat products (e.g. sausages)** |
| v_46 |  | [1]: Ein Teil vom Verkaufspreis wird verwendet, um Projekte zur Verbesserung des Tierwohls zu fördern.  [2]: Ein Teil vom Verkaufspreis wird verwendet, um Projekte zur Unterstützung von Landwirtinnen und Landwirten in der Region zu fördern.  [3]: Ein Teil vom Verkaufspreis wird verwendet, um Projekte zur Reduzierung des ökologischen Fussabdrucks (z.B. CO2 Emissionen) der Lebensmittelproduktion zu fördern.  [4]: Ein Teil vom Verkaufspreis wird verwendet, um Projekte im Bereich der Nachhaltigkeit zu fördern. | [1]: A portion of the sales price is used to support projects to improve animal welfare.  [2]: A portion of the sales price is used to promote projects to support farmers in the region.  [3]: A portion of the sales price is used to support projects to reduce the ecological footprint (e.g. CO2 emissions) of food production.  [4]: A portion of the sales price is used to support sustainability projects. |
|  | **Potato Experiment (mixed-method)** | **Teil 5** | **Part 5** |
|  |  | Im nächsten Teil der Umfrage geht es um Ihre Präferenzen beim Kartoffeleinkauf. | The next part of the survey is about your preferences when buying potatoes. |
|  |  | **FILTER GROUP 1: Frage 1a (Kontrolle / Produzent)** | **FILTER GROUP 1: Question 1a (control / producer)** |
|  |  | Stellen Sie sich vor, Sie wollen festkochende Kartoffeln kaufen.  Im Hofladen finden Sie folgende zwei Produktvarianten. Für welches Produkt entscheiden Sie sich? | Imagine you want to buy waxy potatoes.  You will find the following two product variants in the farm store. Which product do you choose? |
| v_47 |  | Ich wähle:   - Kartoffel A [1] - Kartoffel B [2] - Keine der beiden Kartoffeln [3] | I choose:   - Potato A [1] - Potato B [2] - Neither of the two potatoes [3] |
| v_48 |  | Sie haben sich für ………………. Entschieden. Bitte begründen Sie kurz Ihre Wahl. | You have chosen ................... Please briefly explain your choice. |
|  |  | **FILTER GROUP 2: Frage 1b (Foodwaste Framing / Produzent)** | **FILTER GROUP 2: Question 1b (Foodwaste Framing / Producer)** |
|  |  | Stellen Sie sich vor, Sie wollen festkochende Kartoffeln kaufen.  Im Hofladen finden Sie folgende zwei Produktvarianten. Für welches Produkt entscheiden Sie sich? | Imagine you want to buy waxy potatoes.  You will find the following two product variants in the farm store. Which product do you choose? |
| v_49 |  | Ich wähle:   - Kartoffel A [1] - Kartoffel B [2] - Keine der beiden Kartoffeln [3] | I choose:   - Potato A [1] - Potato B [2] - Neither of the two potatoes [3] |
| v_50 |  | Sie haben sich für ………………. Entschieden. Bitte begründen Sie kurz Ihre Wahl. | You have chosen ................... Please briefly explain your choice. |
|  |  | **FILTER GROUP 3: Frage 1c (Kontrolle / Supermarkt)** | **FILTER GROUP 3: Question 1c (control / supermarket)** |
|  |  | Stellen Sie sich vor, Sie wollen festkochende Kartoffeln kaufen.  Im Supermarkt finden Sie folgende zwei Produktvarianten. Für welches Produkt entscheiden Sie sich? | Imagine you want to buy waxy potatoes.  You will find the following two product variants in the supermarket. Which product do you choose? |
| v_51 |  | Ich wähle:   - Kartoffel A [1] - Kartoffel B [2] - Keine der beiden Kartoffeln [3] | I choose:   - Potato A [1] - Potato B [2] - Neither of the two potatoes [3] |
| v_52 |  | Sie haben sich für ………………. Entschieden. Bitte begründen Sie kurz Ihre Wahl. | You have chosen ................... Please briefly explain your choice. |
|  |  | **FILTER GROUP 4: Frage 1d (Foodwaste Framing / Supermarkt)** | **FILTER GROUP 4: Question 1d (Foodwaste Framing / Supermarket)** |
|  |  | Stellen Sie sich vor, Sie wollen festkochende Kartoffeln kaufen.  Im Supermarkt finden Sie folgende zwei Produktvarianten. Für welches Produkt entscheiden Sie sich? | Imagine you want to buy waxy potatoes.  You will find the following two product variants in the supermarket. Which product do you choose? |
| v_53 |  | Ich wähle:   - Kartoffel A [1] - Kartoffel B [2] - Keine der beiden Kartoffeln [3] | I choose:   - Potato A [1] - Potato B [2] - Neither of the two potatoes [3] |
| v_54 |  | Sie haben sich für ………………. Entschieden. Bitte begründen Sie kurz Ihre Wahl. | You have chosen ................... Please briefly explain your choice. |
|  | **Personal Attitudes** | **Teil 6** | **Part 6** |
|  | Perception of farmers | Nun folgen noch ein paar abschliessende Fragen zu Ihren Einstellungen. | Finally, we would like to ask you a few questions about your attitudes. |
|  |  | **Bitte geben Sie für die nachfolgenden Aussagen an, wie sehr Sie diesen jeweils zustimmen.**  *Stimme gar nicht zu (1) – weder noch (4) – stimme voll und ganz zu (7)* | **Please indicate how strongly you agree with each of the following statements.**  *Strongly disagree (1) - Neither (4) - Strongly agree (7)* |
| v_58 |  | Ich bin Landwirtinnen und Landwirten gegenüber generell positiv eingestellt. | I am generally positive towards farmers. |
| v_59 |  | Die Arbeit der Landwirtinnen und Landwirte ist wichtig und wertvoll für die Gesellschaft. | Farmers’ work is important and valuable for society. |
| v_60 |  | Landwirtinnen und Landwirte setzen sich für das Tierwohl ein. | Farmers are committed to animal welfare. |
| v_61 |  | Landwirtinnen und Landwirte haben ein grosses Umweltbewusstsein. | Farmers have a great environmental awareness. |
| v_62 |  | Bäuerliche Familienbetriebe sind wichtig und sollten erhalten bleiben. | Family farms are important and should be preserved. |
| v_63 |  | Die Arbeit der Landwirtinnen und Landwirte, um die einzigartige Schweizer Landschaft zu erhalten, ist unzureichend. | The work of farmers to preserve the unique Swiss landscape is insufficient. |
| v_64 |  | Der positive Beitrag der Landwirtinnen und Landwirte zu lokalen Gemeinschaften (z.B. lokales Zusammenleben) ist begrenzt. | The positive contribution of farmers to local communities (e.g. local coexistence) is limited. |
| v_65 |  | Die Rolle der Landwirtinnen und Landwirte, um eine gesunde Ernährung der Bevölkerung sicherzustellen, ist fragwürdig. | The role of farmers in ensuring a healthy diet for the population is questionable. |
| v_66 |  | Ein Leben in der Landwirtschaft ist anspruchsvoll und erfordert viel Engagement und Durchhaltevermögen. | Life in agriculture is demanding and requires a great deal of commitment and perseverance. |
| v_67 |  | Für die Landwirtinnen und Landwirte hat der ökonomische/finanzielle Gewinn Vorrang vor allen anderen Überlegungen. | For farmers, economic/financial profit takes precedence over all other considerations. |
|  | **Health consciousness** | **Bitte geben Sie für die nachfolgenden Aussagen an, wie sehr Sie diesen jeweils zustimmen. (Dohle et al., 2014)**  *Stimme überhaupt nicht zu (1) --- stimme voll und ganz zu (6)* | **Please indicate how strongly you agree with each of the following statements. (Dohle et al., 2014)**  *Strongly disagree (1) --- Strongly agree (6)* |
| v_68 |  | Mir ist es wichtig, dass ich mich gesund ernähre. | My health is dependent on how and what I eat. |
| v_69 |  | Meine Gesundheit ist abhängig davon, wie und was ich esse. | I am prepared to leave a lot, to eat as healthy as possible. |
| v_70 |  | Wenn man gesund isst, wird man weniger krank. | If one eats healthily, one gets ill less frequently. |
| v_71 |  | Ich bin bereit, auf Vieles zu verzichten, um möglichst gesund zu essen. | I think it is important to eat healthily. |
|  | **Environmental Attitudes Inventory (EAI)**  **Scale 4** | **Bitte geben Sie für die nachfolgenden Aussagen an, wie sehr Sie diesen jeweils zustimmen.**  [EAI, scale 4 (Milfont & Duckitt, 2010)]  *Stimme überhaupt nicht zu (1) --- stimme voll und ganz zu (7)* | **Please indicate how strongly you agree with each of the following statements.**  [EAI, scale 4 (Milfont & Duckitt, 2010)]  *Strongly disagree (1) --- Strongly agree (7)* |
| v_73 |  | Das Beste am Recycling ist, dass es Geld spart. | One of the best things about recycling is that it saves money |
| v_74 |  | Das Schlimmste am Verlust des Regenwaldes ist, dass dadurch die Entwicklung neuer Medikamente eingeschränkt wird. | The worst thing about the loss of the rainforest is that it will restrict the development of new medicines. |
| v_75 |  | Einer der wichtigsten Gründe, Seen und Flüsse sauber zu halten, ist, dass die Menschen dort Wassersport treiben können | One of the most important reasons to keep lakes and rivers clean is so that people have a place to enjoy water sports |
| v_76 |  | Die Natur ist wichtig, weil sie zur Freude und zum Wohl der Menschen beitragen kann. | Nature is important because of what it can contribute to the pleasure and welfare of humans |
| v_77 |  | Was mich an der Abholzung am meisten beunruhigt, ist, dass es nicht genug Holz für zukünftige Generationen geben wird. | The thing that concerns me most about deforestation is that there will not be enough lumber for future generations. |
| v_78b2a |  | Wir sollten die Umwelt für das Wohl von Pflanzen und Tieren und nicht für das Wohl der Menschen schützen. | We should protect the environment for the well being of plants and animals rather than for the welfare of humans. |
| v_5f90b |  | das menschliche Glück und die menschliche Fortpflanzung weniger wichtig sind als ein gesunder Planet | Human happiness and human reproduction are less important than a healthy planet |
| v_80 |  | Naturschutz ist wichtig, auch wenn er den Lebensstandard der Menschen senkt. | Conservation is important, even if it lowers people's standard of living. |
| v_81 |  | Wir müssen Flüsse und Seen sauber halten, um die Umwelt zu schützen, und NICHT als Orte, an denen Menschen Wassersport betreiben können | We need to keep rivers and lakes clean in order to protect the environment and NOT as places for people to enjoy water sports |
| v_82 |  | Wir sollten die Umwelt schützen, auch wenn das Wohl der Menschen darunter leidet. | We should protect the environment, even if it means people’s welfare will suffer. |
|  | **Environmental Attitudes Inventory (EAI)**  **Scale 8** | **Bitte geben Sie für die nachfolgenden Aussagen an, wie sehr Sie diesen jeweils zustimmen.**  [EAI, scale 8 (Milfont & Duckitt, 2010)]  *Stimme überhaupt nicht zu (1) --- stimme voll und ganz zu (7)* | **Please indicate how strongly you agree with each of the following statements.**  [EAI, scale 8 (Milfont & Duckitt, 2010)]  *Strongly disagree (1) --- Strongly agree (7)* |
| v_83 |  | Ich mache mir keine Gedanken, Wasser oder andere natürliche Ressourcen zu sparen | I could not be bothered to save water or other natural resources. (R) |
| v_84 |  | Ich achte darauf, dass die Heizung in meinem Zimmer im Winter nicht zu stark aufgedreht ist. | I make sure that during the winter the heating system in my room is not switched on too high. |
| v_85 |  | In meinem täglichen Leben bin ich einfach nicht daran interessiert, Wasser und/oder Strom zu sparen. | In my daily life I’m just not interested in trying to conserve water and / or power. (R) |
| v_86 |  | Wann immer möglich, dusche ich kurz, um Wasser zu sparen. | Whenever possible, IO take a short shower in order to conserve water. |
| v_87 |  | Ich schalte das Licht immer aus, wenn ich es nicht mehr brauche. | I always switch the light off when I don’t need it on any more. |
| v_88 |  | Ich fahre mit dem Auto, wann immer es mir passt, auch wenn es die Luft verschmutzt | I drive whenever it suits me, even if it does pollute the atmosphere. (R) |
| v_89 |  | Ich versuche, in meinem Alltag Wege zu finden, um Wasser oder Strom zu sparen. | In my daily life I try to find ways to conserve water or power. |
| v_90 |  | Ich gehöre NICHT zu den Menschen, die sich bemühen, die natürlichen Ressourcen zu schonen. | I am NOT the kind of person who makes efforts to conserve natural resources. (R) |
| v_91 |  | Wann immer möglich, versuche ich, natürliche Ressourcen zu sparen. | Whenever possible, I try to save natural resources. |
| v_92 |  | Selbst wenn die öffentlichen Verkehrsmittel effizienter wären als sie sind, würde ich lieber mit dem Auto fahren. | Even if public transportation was more efficient than it is, I would prefer to drive my car. (R) |

|  | **Thank you and end of survey** | **Teil 7: Schluss** | **Survey Part 7: End** |
| --- | --- | --- | --- |
| v_56 |  | Wir sind schon fast am Ende dieser Befragung. Sollten Sie noch weitere Bemerkungen haben, können Sie diese hier notieren.  [Freitextantwort] | We are almost at the end of this survey. If you have any further comments, you can make a note of them here.  [Free text answer] |
|  |  | **Vielen Dank!**  Somit sind wir jetzt am Ende dieser Befragung angelangt.  Wir danken Ihnen ganz herzlich für Ihre wertvolle Teilnahme an dieser Studie.Sie können die Umfrage jetzt schliessen. | **Thank you very much!**   We have now reached the end of this survey.   Thank you very much for your valuable participation in this study.You can now close the survey. |

# **References**

Ammann, J., Mack, G., Irek, J., Finger, R., & El Benni, N. (2023). Consumers’ meat commitment and the importance of animal welfare as agricultural policy goal   *Appetite*, *112*(105010). <https://doi.org/doi.org/10.1016/j.foodqual.2023.105010>

Dohle, S., Hartmann, C., & Keller, C. (2014). Physical activity as a moderator of the association between emotional eating and BMI: evidence from the Swiss Food Panel. *Psychol Health*, *29*(9), 1062-1080. <https://doi.org/10.1080/08870446.2014.909042>

Michel, F., Hartmann, C., & Siegrist, M. (2021). Consumers’ associations, perceptions and acceptance of meat and plant-based meat alternatives. *Food Quality and Preference*, *87*. <https://doi.org/10.1016/j.foodqual.2020.104063>

Milfont, T. L., & Duckitt, J. (2010). The environmental attitudes inventory: A valid and reliable measure to assess the structure of environmental attitudes. *Journal of Environmental Psychology*, *30*(1), 80-94. <https://doi.org/10.1016/j.jenvp.2009.09.001>
